# Genotyping *HLA-DRB1* and *HLA-DQB1* alleles in Japanese patients with normal tension glaucoma

**Published:** 2010-09-15

**Authors:** Misa Suzuki, Akira Meguro, Masao Ota, Eiichi Nomura, Tetsuo Kato, Naoko Nomura, Kenji Kashiwagi, Fumihiko Mabuchi, Hiroyuki Iijima, Kazuhide Kawase, Tetsuya Yamamoto, Makoto Nakamura, Akira Negi, Takeshi Sagara, Teruo Nishida, Masaru Inatani, Hidenobu Tanihara, Makoto Aihara, Makoto Araie, Takeo Fukuchi, Haruki Abe, Tomomi Higashide, Kazuhisa Sugiyama, Takashi Kanamoto, Yoshiaki Kiuchi, Aiko Iwase, Shigeaki Ohno, Hidetoshi Inoko, Nobuhisa Mizuki

**Affiliations:** 1 Department of Ophthalmology and Visual Science Yokohama City University Graduate School of Medicine, Yokohama, Kanagawa, Japan; 2 Department of Legal Medicine, Shinshu University School of Medicine, Matsumoto, Nagano, Japan; 3 Department of Ophthalmology, University of Yamanashi, Faculty of Medicine, Yamanashi, Japan; 4 Department of Ophthalmology, Gifu University Graduate School of Medicine, Gifu, Japan; 5 Department of Surgery, Division of Ophthalmology, Kobe University Graduate School of Medicine, Kobe, Hyogo, Japan; 6 Department of Biomolecular Recognition and Ophthalmology, Yamaguchi University School of Medicine, Ube, Yamaguchi, Japan; 7 Department of Ophthalmology and Visual Science, Graduate School of Medical Sciences, Kumamoto University, Kumamoto, Japan; 8 Department of Ophthalmology, University of Tokyo School of Medicine, Tokyo, Japan; 9 Division of Ophthalmology and Visual Science, Graduated School of Medical and Dental Sciences, Niigata University, Niigata, Japan; 10 Department of Ophthalmology and Visual Science, Kanazawa University Graduate School of Medical Science, Kanazawa, Ishikawa, Japan; 11 Department of Ophthalmology and Visual Science, Graduate School of Biomedical Sciences, Hiroshima University, Hiroshima, Japan; 12 Department of Ophthalmology, Tajimi Municipal Hospital, Tajimi, Gifu, Japan; 13 Department of Ophthalmology, Hokkaido University Graduate School of Medicine, Sapporo, Japan; 14 Department of Genetic Information, Division of Molecular Life Science, Tokai University School of Medicine, Isehara, Kanagawa, Japan

## Abstract

**Purpose:**

Normal tension glaucoma (NTG) is a subtype of glaucoma in which intraocular pressure is within the statistically normal range. NTG may be associated with an immune disorder. The aim of this study was to determine whether specific alleles in the human leukocyte antigen (*HLA*)-*DRB1* and *HLA-DQB1* genes correlated with NTG in Japanese patients.

**Methods:**

We genotyped the *HLA-DRB1* and *HLA-DQB1* alleles in 113 Japanese patients with NTG and in 184 healthy Japanese control subjects using the polymerase chain reaction-sequence-specific oligonucleotide probes (PCR-SSOP) Luminex method. We assessed the allelic diversity in patients and controls.

**Results:**

There were no statistically significant differences in the allele frequency of *HLADRB1* and *HLA-DQB1* between NTG patients and control subjects, and no *HLA-DRB1*-*HLA-DQB1* haplotypes demonstrated any significant association with NTG.

**Conclusions:**

Our findings suggest that *HLA-DRB1* and *HLA-DQB1* polymorphisms have no significant effect on the development of NTG in Japanese patients.

## Introduction

Glaucoma is a progressive optic neuropathy leading to permanent visual loss that affects approximately 70 million people worldwide [1,2]. The most common type of glaucoma is primary open angle glaucoma (POAG). Glaucoma is generally a multifactorial disorder caused by environmental and/or genetic factors. High intraocular pressure (IOP) is the most important risk factor for glaucoma [3] and has previously been used as a diagnostic criterion for the disease. A subset of patients with POAG have normal IOP, a condition called normal tension glaucoma (NTG) [4-6]; thus, factors other than IOP clearly influence the pathogenesis of glaucomatous optic neuropathy. The prevalence of NTG is 3.6% in Japan, which is higher than in other ethnicities [7]. NTG often goes undiagnosed until after vision deteriorates because the symptoms are minimal and IOP is normal. Although relatively higher IOP, myopia, and older age are known to be factors associated with the development of NTG [8], they are not pathognomonic. Other pathogenic factors such as vasospasm, ischemia, and vascular insufficiencies are also indicated to be associated with the development of NTG [9-12].

It was recently suggested that the autoimmune system plays an important role in the development of glaucoma, especially NTG. Wax [13] proposed that the role of the immune system in glaucoma was twofold and that an autoimmune mechanism may elicit damage to the optic nerve, resulting in glaucomatous injury. He additionally suggested that autoimmune-mediated glaucoma injury occurs most often in NTG patients. There have been several reports of autoantibodies against ocular antigens that are upregulated in the serum of patients with glaucoma, such as heat shock proteins (HSPs) [14], gamma-enolase [15], glutathione S-transferase [16], anti-phosphatidylserine [17], and glycosaminoglycans [18]. Therefore, NTG might actually be an organ-specific autoimmune disorder.

Human leukocyte antigen (HLA) genes regulate the immune system and the predisposition to most autoimmune disorders. HLA class II alleles have been shown to be significantly associated with glaucoma. Gil-Carrasco et al. [19] reported a higher frequency of the HLA-DR3 antigen in Mexican POAG patients compared with controls; further, an increased frequency of the HLA-DRB1*0407-DQB1*0302 haplotype was significantly associated with POAG in Mexican patients [20]. Ferreri et al. [21] showed a significant association between DR11 and DQ1 alleles and POAG in the Italian population. Therefore, HLA class II gene polymorphisms may influence the development of autoimmunity in glaucoma. On the other hand, HLA class I alleles have also been shown to be associated with glaucoma [22-24]. However numerous studies have reported no significant correlation between HLA class I alleles and glaucoma [25-28], and thus the possibility of HLA class I alleles being associated with the pathogenesis of NTG is deemed low.

The aim of this study was to clarify the role of HLA class II gene polymorphisms in the development of NTG by assessing the association of polymorphisms in *HLA-DRB1* and *HLA-DQB1* genes with NTG in Japanese patients.

## Methods

### Subjects

We recruited 113 unrelated Japanese patients with NTG and 184 unrelated healthy Japanese control subjects at the following Japanese universities: Yokohama City University, Yamanashi University, Gifu University, Kobe University, Yamaguchi University, Kumamoto University, Hokkaido University, Tokyo University, Niigata University, Kanazawa University, Hiroshima University, Tajimi municipal hospital, and Tokai University. Patients were diagnosed using the following strict inclusion criteria: the presence of glaucomatous optic neuropathy with corresponding visual field loss; normal open angle with angle width of Shaffer grade 2 or higher; absence of IOP greater than 21 mmHg on repeat pressure measurement using Goldmann applanation tonometry without medication; and lack of a pathological basis for optic nerve changes upon neurologic, rhinologic, and general medical examination, including magnetic resonance imaging. Glaucomatous optic nerve change was diagnosed when the vertical cup/disc ratio of the optic nerve head was 0.7 or higher, the rim width at the superior (11 to 1 o’clock) or inferior (5 to 7 o’clock) position was less than or equal to 10% of the disc diameter, the difference in the vertical cup/disc ratio between eyes was 0.2 or greater, or a nerve fiber layer defect was found. Glaucomatous visual field loss was defined on a hemifield basis using reliable field data examined by the Humphrey® static visual field analyzer (HFA) C-30–2 program (Carl Zeiss Meditec, Oberkochen, Germany) according to Anderson and Patella’s criteria [29]. The hemifield was considered abnormal when the pattern deviation probability plot revealed a cluster of 3 or more non-edge contiguous points having sensitivity with a probability of less than 5% in the upper or lower hemifield, with one of these points having a probability of less than 1%. In addition, the following inclusion and exclusion criteria were used. We excluded individuals who were diagnosed when they were under 20 years old or over 60 years old with a –8.0D or higher myopic refractive error of spherical equivalence. The selection criteria for the HFA mean deviation were stratified depending on the subjects’ ages to minimize the effect of aging on retinal ganglion cell loss and subsequent visual field defects. Specifically, there was no exclusion if the patient was diagnosed when under 50 years old; when the patient was –10.00 dB or worse in at least one eye but was diagnosed between the ages of 50 and 55 years; or when the patients had –15.00 dB or worse in at least one eye but the patient was over age 55 when diagnosed. Patients with relatively early onset were selected because early onset suggests a stronger involvement of genetic factors. During diagnosis, patients were excluded if their refraction values had changed due to cataract surgery, refractive surgery, etc. In cases in which glaucomatous visual field loss was present in only one eye, the refraction value and glaucomatous visual field loss of the affected eye were used. In cases in which glaucomatous visual field loss was present in both eyes, the refraction value and glaucomatous visual field loss of the more severely affected eye were used.

Patients ranged in age between 24 and 59 years (mean 47.4±6.9 years), and 47.8% were male. The mean refraction value was –3.82±3.02 D, and the mean deviation observed in the Humphrey® static visual field determination (Carl Zeiss Meditec) was –10.15±8.01 dB. The control subjects were healthy volunteers from a geographic region similar to that of the NTG patients. They had no glaucoma or ophthalmological or systemic diseases that might cause glaucoma or optic nerve changes, and they had either no myopia or mild myopia with refractive errors of –3.00 D or less. Of the control subjects, 44.6% were male.

### *HLA-DRB1* and *HLA-DQB1* genotyping

Genotyping of the *HLA-DRB1* and *HLA-DQB1* alleles was performed using the PCR-SSOP Luminex method using LABType SSO (One Lambda Inc., Canoga Park, CA), a reverse SSO DNA typing system, according to the manufacturer’s instructions. Briefly, target DNAs were PCR-amplified using *HLA-DRB1* or *HLA-DQB1* group-specific primers. The biotinylated PCR products were denatured and hybridized to locus-specific probes conjugated to fluorescently coded microspheres. A flow analyzer, LABScanTM 100, identifies the fluorescent intensity of phycoerythrin on each microsphere. The determination of the HLA allele was based on the reaction pattern compared to patterns associated with published HLA allele sequences.

### Statistical analysis

Allelic frequencies were determined by direct counting. The significance of the allelic distribution between the patients with NTG and normal controls was first analyzed using Fisher’s exact probability test. The probability of association was corrected by the Bonferroni inequality method, i.e., by multiplying the p values with the number of alleles. A corrected p (pc) value of <0.05 was considered statistically significant.

Haplotype frequencies and linkage disequilibrium (LD) in the multi-locus analyses were calculated using PyPop [30]. Haplotype frequencies were determined using the iterative expectation-maximization algorithm. LD was measured using Hedrick’s multiallelic D´ statistic [31]. D´ weights the contribution of specific allele pairs to the LD using the product of their allelic frequencies. The measure is normalized to fall between zero and one, with higher values indicating a stronger LD contribution.

## Results

The gene frequencies of *HLA-DRB1* and *HLA-DQB1* alleles in 113 Japanese patients with NTG and in184 healthy control subjects are shown in Table 1 and Table 2, respectively. A total of 27 *HLA-DRB1* alleles and 14 *HLA-DQB1* alleles were identified in NTG patients and healthy controls. The frequencies of HLA-DRB1*1301 and HLA-DQB1*0603 were higher in patients with NTG compared with healthy controls, although this increase did not reach statistical significance after performing the Bonferroni correction. We found no significant differences in the frequency of other *HLA-DRB1* and *HLA-DQB1* alleles between the patients and controls.

**Table 1:** Allele frequencies of *HLA-DRB1* in Japanese patients with normal tension glaucoma.

** **	**Frequency, n (%)**		** **	** **
***HLA-DRB1***	**Cases (n=113)**	**Controls (n=184)**	**p**	**pc**
0101	17 (7.5)	20 (5.4)	0.38	** **
0301	0 (0.0)	1 (0.3)	1	** **
0401	0 (0.0)	1 (0.3)	1	** **
0403	6 (2.7)	12 (3.3)	0.81	** **
0405	27 (11.9)	43 (11.7)	1	** **
0406	5 (2.2)	11 (3.0)	0.79	** **
0407	1 (0.4)	3 (0.8)	1	** **
0410	8 (3.5)	7 (1.9)	0.28	** **
0802	12 (5.3)	23 (6.3)	0.72	** **
0803	15 (6.6)	30 (8.2)	0.53	** **
0901	30 (13.3)	54 (14.7)	0.72	** **
1001	2 (0.9)	0 (0.0)	0.14	** **
1101	5 (2.2)	8 (2.2)	1	** **
1201	9 (4.0)	12 (3.3)	0.65	** **
1202	7 (3.1)	8 (2.2)	0.59	** **
1301	5 (2.2)	1 (0.3)	0.032	NS
1302	16 (7.1)	28 (7.6)	0.87	** **
1303	0 (0.0)	1 (0.3)	1	** **
1307	0 (0.0)	1 (0.3)	1	** **
1401	7 (3.1)	13 (3.5)	1	** **
1403	1 (0.4)	5 (1.4)	0.42	** **
1405	2 (0.9)	7 (1.9)	0.49	** **
1406	4 (1.8)	3 (0.8)	0.44	** **
1412	0 (0.0)	1 (0.3)	1	** **
1501	17 (7.5)	23 (6.3)	0.61	** **
1502	28 (12.4)	48 (13.0)	0.90	** **
1602	2 (0.9)	4 (1.1)	1	** **

**Table 2:** Allele frequencies of *HLA-DQB1* in Japanese patients with normal tension glaucoma.

** **	**Frequency, n (%)**		** **	** **
***HLA-DQB1***	**Cases (n=113)**	**Controls (n=184)**	**p**	**pc**
0201	0 (0.0)	1 (0.3)	1	** **
0301	22 (9.7)	37 (10.1)	1	** **
0302	21 (9.3)	40 (10.9)	0.58	** **
0303	35 (15.5)	57 (15.5)	1	** **
0401	26 (11.5)	40 (10.9)	0.89	** **
0402	14 (6.2)	20 (5.4)	0.72	** **
0501	19 (8.4)	21 (5.7)	0.24	** **
0502	5 (2.2)	10 (2.7)	0.79	** **
0503	6 (2.7)	15 (4.1)	0.49	** **
0601	42 (18.6)	77 (20.9)	0.53	** **
0602	14 (6.2)	21 (5.7)	0.86	** **
0603	5 (2.2)	1 (0.3)	0.032	NS
0604	16 (7.1)	28 (7.6)	0.87	** **
0611	1 (0.4)	0 (0.0)	0.38	** **

The magnitude of LD between *HLA-DRB1* and *HLA-DQB1* was extremely high, with a pair-wise D´>0.85. Haplotype analyses indicated complete LD between the HLADRB1*1301 and HLA-DQB1*0603 alleles, and the HLA-DRB1*1301-HLA-DQB1*0603 haplotype had an increased frequency in patients; this was not significant after correction (Table 3).

**Table 3:** Frequencies of DRB1-DQB1 haplotypes in Japanese patients with normal tension glaucoma (NTG).

** **	**Frequency, n (%)**		** **	** **
Haplotype*	**Cases (n=113)**	**Controls (n=184)**	**p**	**pc**
DRB1*0901:DQB1*0303	29 (12.8)	53 (14.4)	0.63	** **
DRB1*1502:DQB1*0601	28 (12.4)	47 (12.8)	1	** **
DRB1*0405:DQB1*0401	26 (11.5)	40 (10.9)	0.89	** **
DRB1*1302:DQB1*0604	16 (7.1)	28 (7.6)	0.87	** **
DRB1*0803:DQB1*0601	14 (6.2)	29 (7.9)	0.52	** **
DRB1*0101:DQB1*0501	17 (7.5)	20 (5.4)	0.38	** **
DRB1*1501:DQB1*0602	14 (6.2)	19 (5.2)	0.59	** **
DRB1*1301:DQB1*0603	5 (2.2)	1 (0.3)	0.032	NS

*Only haplotypes that reached frequencies >5% in all subjects or that may have an effect on the development of NTG are shown.

## Discussion

Several studies have reported associations between HLA and glaucoma, and others have suggested that HLA class II genes may have an effect on the development of POAG [19-21]. The aim of this study was to investigate whether specific alleles in the HLA class II genes affected the development of NTG, which was recently suggested to be an autoimmune disorder. Toward this end, we genotyped the *HLA-DRB1* and *HLA-DQB1* genes in Japanese NTG patients and in healthy control subjects.

In contrast to previous POAG studies, this study focused on NTG and did not find an allelic distribution of HLA class II genes that was statistically different between NTG patients and controls. Although the frequencies of the HLA-DRB1*1301 and HLA-DQB1*0603 alleles and the HLA-DRB1*1301-HLA-DQB1*0603 haplotype was higher in NTG patients, these increases were not statistically significance after the Bonferroni correction. These HLA types were not consistent with POAG-associated HLA types. In addition, the frequency of POAG-associated HLA types was not significantly different in patients and controls in this NTG study, and these HLA types are rare in the Japanese population.

Our NTG study did not replicate the results of previous POAG studies, most likely because NTG and POAG have distinct characteristics. The genes affecting the immune systems of NTG patients may differ from those affecting POAG patients. In addition, the distribution of the alleles of HLA genes is different for each ethnic group. It is possible that there were false-positive results in the earlier POAG studies: In HLA disease association studies, careful study design is required to observe reliable positive associations. Further, the studies must be performed using adequate sample sizes, careful multiple testing correction, and LD analysis. Although Gil-Carrasco et al. [19] and Ferreri et al. [21] reported the associations of HLA class II alleles with POAG, the sample sizes in those studies were not big enough to support their positive findings, since positive results in small studies have a high risk of being false-positives. In another study that reported a significant association between the HLA-DRB1*0407-HLA-DQB1*0302 haplotype and POAG [20], the p values were corrected by the Yates method but not by multiple testing; this, too, might lead to a false positive association. In addition, the study reported that the estimated frequency for the HLA-DRB1*0407- HLA-DQB1*0302 haplotype in the POAG patients was 25.9%, much higher than the 15.4% frequency reported for the HLA-DRB1*0407 allele in the patients. Based on the allele frequency shown in the study, it is possible that actual frequency of the haplotype was less than estimated and that the haplotype may be not associated with POAG. Recently, Yano et al. [32] reported a possible association between the HLA-DRB1*1502 allele and the presence of anti-neurofilament light subunit antibodies in a patient group that included POAG and NTG patients; no correction was performed. However, if the Bonfferoni correction is applied to that study, the adjusted p value would be greater than 0.05.

It thus remains unclear whether the HLA class II genes have an effect on the pathogenesis of glaucoma. Several clinical and experimental glaucoma studies have focused on the role of the immune system in glaucoma; results strongly suggest that activated immunity contributes to glaucomatous optic neuropathy [33]. Cartwright et al. [34] reported that 30% of NTG patients had one or more immune-related diseases compared to 8% of control patients with ocular hypertension. This suggests that the immune system plays a role in the development of NTG. It is likely that multiple immune-related genes are associated with NTG. For example, the toll-like receptor 4 gene encodes a protein that recognizes heat shock proteins and lipopolysaccharides and is critical for the innate and adaptive immune systems. We recently reported that polymorphisms in this gene affected the development of NTG [35]. However, there are no reports of a significant association between other immune-related genes and NTG. In the future, carefully designed studies will be essential to determine whether other immune-related genes contribute to the pathogenesis of NTG.

In conclusion, we found no significant associations between the genes *HLA-DRB1* and *HLA-DQB1* and NTG in the Japanese population. These genes do not appear to be associated with the pathogenesis of NTG. However, because there are differences in the distribution of alleles of HLA genes in different ethnic groups, the association between genes and NTG should also be assessed in other ethnic groups.
